# Responsiveness and minimum important change of the Oswestry Disability Index in Italian subjects with symptomatic lumbar spondylolisthesis

**DOI:** 10.1007/s10195-017-0446-y

**Published:** 2017-02-16

**Authors:** Carla Vanti, Silvano Ferrari, Jorge Hugo Villafañe, Pedro Berjano, Marco Monticone

**Affiliations:** 1 Department of Molecular Medicine, University of Padova, Padua, Italy; 2 IRCCS Don Gnocchi Foundation, Milan, Italy; 3 IRCCS Istituto Ortopedico Galeazzi, Milan, Italy; 4 Department of Public Health, Clinic and Molecular Medicine, University of Cagliari, Cagliari, Italy; 5 Via Tosarelli 144, Castenaso, 40055 Bologna, Italy

**Keywords:** Spondylolisthesis, Low back pain, Responsiveness, Oswestry Disability Index, Outcome measures

## Abstract

**Background:**

This study aims to investigate the responsiveness and the minimum important change of the Italian version of the Oswestry Disability Index (ODI-I) in subjects with symptomatic specific low back pain associated with lumbar spondylolisthesis (SPL).

**Materials and methods:**

One hundred and fifty-one patients with symptomatic SPL completed the ODI-I, a 0–100 numerical rating scale (NRS), and performed the prone and supine bridge tests. The global perception of effectiveness was measured with a 7-point Likert scale. Responsiveness was assessed by distribution methods (minimum detectable change [MDC], effect size [ES], standardized response mean [SRM]) and anchor-based methods (ROC curves).

**Results:**

The MDC was 4.23, the ES was 0.95 and the SRM was 1.25. ROC analysis revealed an area under the curve of 0.76 indicating moderate discriminating capacity. The best cut-off point for the dichotomous outcome was 7.5 (sensitivity 90.3%, specificity 56.7%). .

**Conclusions:**

The ODI-I proved to be responsive in detecting changes after conservative treatment in subjects with lumbar SPL.

**Level of evidence:**

II.

## Introduction

The ability of a scale to be sensitive to change (responsiveness) is important not only in the clinical setting, but also for research, allowing power calculations, sample size estimates and cost evaluations [1]. When a scale is applicable on a wide range of clinical conditions, it is necessary to investigate whether the baseline scores and the change in scores are similar on the various categories of subjects to which the scale is administered or, conversely, whether the different diagnostic subgroups show dissimilar results [2].

The Oswestry Disability Index (ODI) is one of the most widely used questionnaires measuring low back pain (LBP)-related disability [3]. It is a self-administered 10-item questionnaire, composed by one section rating the intensity of pain and nine others describing the disabling effect of LBP on daily activities. The score for each item ranges from 0−5, and the sum of the ten scores is expressed as a percentage of the maximum score, ranging from 0 (no disability) to 100 (maximum disability). The values proposed for the minimum important change of ODI are a reduction of 10 points, or a decrease of 30% compared to the initial value [4].

The ODI has been translated and culturally adapted into several languages, including Italian [5], and its responsiveness was investigated in Italian subjects complaining of subacute and chronic non-specific LBP. The minimal detectable change (MDC) for the ODI was 13.67, the effect size (ES) was 0.53, and the standardized response mean (SRM) was 0.80. The best cut-off point for the dichotomous outcome was 9.5 (sensitivity 76%, specificity 63%). ROC analysis revealed an area under the curve of 0.71. ODI moderately correlated with the numerical rating scale (NRS). These results were consistent with other published studies on non-specific LBP [6].

International literature investigated the psychometric properties of the ODI in different LBP subgroups, including spondylolisthesis (SPL). The weighted main ODI score in SPL was calculated on 120 subjects (pooled data from different studies) as 26.63, and the weighted mean difference as 14.4 [2]. Nevertheless, these pooled data came from studies conducted in very different settings—four studies on surgically treated patients [7–10], and one study involving 44 subjects on conservatively treated patients [11].

To the best of our knowledge, there is only one other study on the responsiveness of the ODI in a conservative setting for SPL—a non-randomized trial of 20 patients in which the main ODI score in SPL was 30.35 and the mean difference was 10.20 [12].

No study has been conducted on the Italian version of the ODI (ODI-I) in clinical conditions different from non-specific LBP. The objective of this study is to examine the responsiveness and the minimum important change of the ODI-I in Italian subjects with symptomatic specific LBP associated with lumbar SPL undergoing a physical therapy program.

## Materials and methods

### Design

A prospective cohort observational study was conducted. The present paper was prepared according to the editorial form of medical publishing and STROBE publishing rules [13].

### Participants

A total of 151 subjects with symptomatic lumbar SPL were diagnosed according to the gold criteria [14], by the referring orthopedic doctors or spinal surgeons. Before starting the conservative treatment, the patients were informed about the different therapeutic options by their specialist, and a shared decision was reached. All patients complained of LBP, namely pain, muscle tension, or stiffness localized below the costal margin and above the inferior gluteal folds, with or without sciatica.

All patients were clinically stable and they all underwent a physical examination by two physical therapists with expertise in orthopedic manual therapy. The examiners verified the presence of Waddell’s signs, which are suggested by Italian LBP Guidelines to exclude the presence of non-organic pain, due to a major psychological or social involvement [15].

The inclusion criteria were a diagnosis of symptomatic SPL, aged >18 years, a diagnosis of SPL confirmed by X-ray, CT or MRI, level L4/L5 or L5/S1, isthmic or degenerative types [15], and the ability to speak and write in Italian. Subjects who had undergone previous lumbar surgery, who were affected by systemic diseases (e.g., inflammatory or infectious pathologies, cancer, etc.), spinal specific pathologies (e.g., spinal stenosis, inflammatory spinal diseases such as ankylosing spondylitis, discitis, and arachnoiditis), neuromuscular disorders, cognitive deficits, or who did not sign the informed consent were excluded.

### Outcome measures

Two physical therapists with expertise in orthopedic manual therapy collected the measurements and administered the treatments. The NRS [16] and the ODI-I [5] were administered to measure pain and disability, respectively. Subjects also completed a global perception effect (GPE) questionnaire. This questionnaire is a 7-point Likert-type scale comprising only one question to evaluate the subject’s self-reported improvement or deterioration after the intervention. Two clinical tests commonly used to detect muscle endurance were performed—the prone bridge test (PBT) and the supine bridge test (SBT) [17].

### Procedures

The patients signed the informed consent, provided demographic and clinical data, and completed the ODI-I and the NRS. A specific schedule was prepared to collect main comorbidities. All the forms were placed in a closed envelope. The physical therapists then asked the subjects to perform the bridge tests, and the results were recorded on a separate form.

The SBT was performed in the supine position, asking the subject to raise his/her pelvis from the table so that his/her shoulders, hips, and knees were maintained in a straight line. The PBT was performed in the prone position, asking the subject to raise his/her pelvis from the table so that only his/her forearms and his/her toes were in contact with the table. These positions were sustained until fatigue or pain prevented the maintenance of the test position and the physical therapists recorded the holding time in seconds.

Patients attended physical therapy treatment for a number of sessions and over a period depending on the individual patient’s needs. Each session lasted 1 h, and included supervised exercises and home exercises aiming to improve lumbar stability, according to the therapeutic program proposed by Richardson et al. [18].

Progressively, the exercises involved all lumbar muscles, increasing range of movement, load and speed and advancing towards more complex movement patterns, and maintaining muscle stabilization. This program was found to be effective in a previous study [19].

A functional and graded approach was also performed to increase activity level and improve strength, endurance, range of motion, balance, and coordination [20].

Immediately at the end of the treatment, patients completed the ODI-I, NRS and GPE questionnaires, which were placed in a closed envelope, and physical therapists recorded the results of the bridge tests. Post-treatment testing was performed by the same assessor who carried out the pre-treatment measurements. The administrative staff created an electronic database with the collected data.

### Statistical analysis

Data were analyzed using SPSS version 21.0 (SPSS Inc, Chicago, IL, USA).

Responsiveness was assessed by means of distribution methods—MDC, ES, SRM and anchor-based methods (ROC curves) [20]. Spearman’s rank correlation coefficient (*R*_s_) was used to evaluate the relationship between the ODI and other parameters evaluated. The *R*_s_ values were interpreted according to Domholdt’s recommendations.

Statistical analysis was conducted at a 95% confidence level, and *P* < 0.05 was considered statistically significant.

## Results

**Fig. 1:**
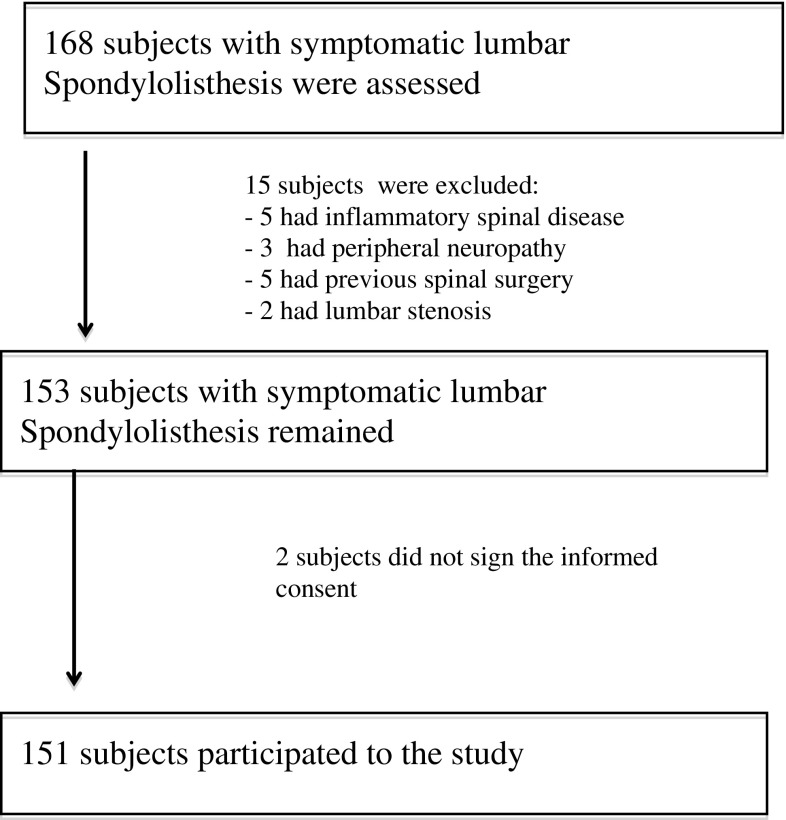
Flow chart

One hundred and sixty-eight consecutive subjects with symptomatic SPL were screened for eligibility criteria. One hundred and fifty-one satisfied all eligibility criteria and agreed to participate (Fig. 1). The mean age was 45 ± 15 years, with 62.9% women. The mean number of sessions was 8 ± 2 and the mean duration of the treatment was 2 ± 1 months. The characteristics of the sample are shown in Table 1. All subjects attended the treatment sessions and completed the follow-up.

**Table 1:** Characteristics of the sample

Variable	Category	*N*	%
Age (years) in classes	18–24	19	12.7
	25–29	11	7.3
	30–34	13	8.7
	35–39	16	10.7
	40–44	17	11.3
	45–49	12	8.0
	50–54	15	10.0
	55–59	13	8.7
	60–64	17	11.3
	>65	17	11.3
Marital status	Married	91	60.3
	Unmarried	60	39.7
Work activity	Student	16	10.6
	Employee	65	43.0
	Self-employed	26	17.2
	Retired	24	15.9
	Houseworker	19	12.6
	Unemployed	1	0.7
Education	Elementary school	4	2.6
	Mid-school	13	8.6
	Upper school	74	49.0
	University	60	39.7
Smoker	Yes	32	21.2
	No	119	78.8
Level of spondylolisthesis	L5/S1	113	74.8
	L4/L5	36	23.8
	L4/L5 and L5/S1	2	1.3
Type of spondylolisthesis	Isthmic	102	67.5
	Degenerative	49	32.5
Grade of spondylolisthesis	I	123	81.5
	II	27	17.9
	III	1	0.7
	IV	–	–
Pain duration in months	0–1	22	14.6
	2–3	22	14.6
	>3	107	70.9
Referred pain	Yes	74	49.3
	No	76	50.7
Drugs^a^	Antidepressants	7	4.6
	Analgesics	29	19.2
	NSAIDs^b^, steroids	12	8
	Muscle relaxants	1	0.7
Comorbidities^c^	Heart disease	4	2.6
	Respiratory disease	2	1.3
	Enteric disease	8	5.2
	Endocrinal disease	2	1.3
	Renal disease	1	0.7
	Orthopedic dysfunction	9	5.9
	Anxiety/depression	9	5.9

^a^Some patients took more than one drug

^b^ *NSAIDs* non-steroidal anti-inflammatory drugs

^c^Some patients had more than one comorbidity

The main ODI score at the beginning of the treatment was 22.8 ± 12.9 and the main post-treatment change was −10.7 ± 0.9. All other outcome measures (NRS, PBT, and SBT) showed statistically significant improvements after the period of treatment (Table 2).

**Table 2:** Instruments scores before and after treatment

Method	*N*	Mean ± standard deviations (SD)		
		Pre-treatment	Post-treatment	Difference within groups
NRS				
Improved	120	42.6 ± 21.8	14.5 ± 16.0	−28.1 ± 20.4
Not improved	31	52.4 ± 20.0	39.5 ± 19.5	−12.9 ± 20.0
Total	151	44.6 ± 21.7	19.6 ± 19.5	−25.0 ± 20.6
ODI-I				
Improved	120	22.2 ± 13.1	9.7 ± 8.5	−12.5 ± 10.0
Not improved	31	23.2 ± 14.5	21.1 ± 16.3	−2.2 ± 13.1
Total	151	22.4 ± 12.0	12.0 ± 11.4	−10.4 ± 10.4
PBT				
Improved	120	21.7 ± 25.2	43.0 ± 32.8	21.2 ± 23.4
Not improved	31	14.4 ± 20.0	20.0 ± 23.8	5.6 ± 9.6
Total	151	20.2 ± 24.7	38.3 ± 32.4	18.0 ± 19.6
SBT				
Improved	120	76.8 ± 60.2	125.4 ± 54.8	48.6 ± 50.6
Not improved	31	56.3 ± 54.8	70.7 ± 59.0	14.5 ± 27.5
Total	151	72.6 ± 59.6	114.2 ± 59.7	41.6 ± 50.0

Data are expressed as means ± standard deviations (SD)

*N* number, *ODI-I* Oswestry Disability Index (Italian version), *NRS* numerical rating scale, *PBT* prone bridge test, *SBT* supine bridge test

Sixty-eight (45%) of the subjects reported ‘completely better’, 52 (34.4%) reported ‘much better’ only, and the remaining 31 (20.6%) reported both ‘little better’ and ‘about the same’.

The MDC was 4.23, the ES was 0.95 and the SRM was 1.25; ROC analysis revealed an area under the curve of 0.76 indicating moderate discriminating capacity. The best cut-off point for the dichotomous outcome was 7.5 (sensitivity 90.3%, specificity 56.7%) (Table 3).

**Table 3:** Responsiveness of NRS and ODI-I

Method	Value	Improved	Not improved
	Total		
NRS			
Minimum detectable change	9.77	7.98	9.75
Effect size	1.15	1.29	0.65
Effect size (Guyatt)	1.25	1.41	0.65
Standardized response mean	1.21	1.38	0.65
Optimal cut-off point (AUC, sensitivity, specificity)	17.5 (0.85, 90.3, 37.5)		
ODI-I			
Minimum detectable change	5.72	4.23	8.14
Effect size	1.0	0.95	0.15
Effect size (Guyatt)	0.87	0.86	0.15
Standardized response mean	1.0	1.25	0.17
Optimal cut-off point (AUC, sensitivity, specificity)	7.5 (0.76, 90.3, 56.7)		

*ODI-I* Oswestry Disability Index (Italian version), *NRS* numerical rating scale

Spearman’s rank correlation coefficients showed a moderately, significant and negative relationship between the ODI-I and PBT and SBT (*R*_s_ = −0.5 and −0.48, respectively, both *P* < 0.001) and a good, significant and direct relationship between the ODI-I and the NRS (*R*_s_ = 0.62, *P* < 0.001) (Table 4).

**Table 4:** Spearman’s rank correlation coefficients between the ODI-I and other parameters

Basal metabolic data	Spearman’s *r*	*P* value
Age	0.21	0.001
NRS	0.62	0.001
GPE	0.36	0.001
PBT	−0.45	0.001
SBT	−0.48	0.00

*NRS* numerical rating scale, *GPE* global perceived effect, *PBT* prone bridge test, *SBT* supine bridge test

## Discussion

This study investigated the responsiveness and the minimum important change of the ODI-I on a sample of 151 SPL patients who attended a physical therapy program. The main ODI-I score (22.8) and the main post-treatment changes (−10.7) were similar although slightly lower than those calculated by Fairbank and Pynsent (26.6 and 14.4, respectively) in SPL subjects [2]. The best cut-off point (−7.5) was lower than that found in a previous study on ODI-I in non-specific LBP (−9.5) [6], whereas the AUCs are similar (0.76 and 0.71, respectively), suggesting moderate discriminating ability of this questionnaire.

The changes in ODI-I scores are in line with the values proposed for the minimum important change by Ostelo et al. [4], i.e., a reduction of 10 points, or a decrease of 30% compared to baseline.

Our results showed a relevant and comparable effect of the treatment on the ODI-I score. The changes in ODI-I score also appeared significantly related to the amount of perceived improvement and were coherent with the changes in the other outcome measures concerning lumbar pain and muscular endurance. The correlation between pain change scores and ODI change scores is in line with a previous study on patients submitted to spinal surgery [21]. Unfortunately, we cannot comment about the correlation with bridge tests due to the lack of published studies on this topic.

Our findings should be analyzed in the light of some factors that can affect the results of outcome measures in LBP. First, we consider that the expectation of improvements and the stage of the pain can influence the rates of change which are higher in acute compared to chronic subjects [22]. Moreover, due to the multifactorial origin of the LBP, we cannot exclude that lumbar disc derangements or other dysfunctions instead of SPL caused pain [24].

Furthermore, both the variability within the population and the inter-individual differences can influence the responsiveness of a measure. As observed by Lauridsen et al. [23], an increase of 25% in ODI baseline score provokes a 12-point augmentation in the minimum important change of this measure. Demoulin et al. [24] also stressed the relevance of the variability of the time between evaluations on the responsiveness of a measure. In our sample, duration of the pain was variable and questionnaires were administered only at baseline and immediately after the treatment, without any further follow-up. This suggests caution in interpreting the results.

### Study limitations

The limitations of this study can be related to the failure of the patient to assess the change, which could also be reflected in the final disability score. It can cause measurement errors on global evaluation and errors on disability assessment as correlated. Moreover, a one-question global assessment score may not differentiate between quantitative and qualitative perception of change [25].

Other limitations are related to the execution of clinical tests [26], because the same physical therapists performed the clinical tests and conducted the treatments.

As most of the patients reported a better condition on GPE, we cannot comment about the responsiveness of the ODI-I in subjects who reported a worsened state.

The results of this study cannot be applied to different categories of specific LBP, because our inclusion criteria only selected subjects with lumbar SPL.

This series included only patients treated non-operatively (as definitive treatment or as an initial attempt before proceeding with an indication of surgical treatment). Thus, our findings could not be applicable to those patients who having more severe forms of spondylolisthesis needed surgical treatment first. Finally, as our sample included both isthmic and degenerative spondylolisthesis, we cannot draw any conclusion about a difference in responsiveness between these two groups.

Suggestions for future studies are to investigate the responsiveness of the ODI-I in various LBP subgroups, submitted to different treatments or assessed with other outcome measures.

In conclusion, this study demonstrated a moderate responsiveness of the ODI-I in detecting clinical changes after physical therapy treatment in subjects with symptomatic specific LBP associated to lumbar SPL. These findings are coherent with those published in the literature with different LBP samples.
